# The Curability of Morphia Addiction

**Published:** 1888-08

**Authors:** 


### THE CURABILITY OF MORPHIA ADDICTION.

Those that are familiar with the most rational methods of treating the morphine habit are prepared to give a favorable prognosis in a majority of cases, but there are many physicians, intelligent in general professional subjects, whose attention has never been carefully directed to narcotic inebriety, and who therefore entertain grave doubts of curing the confirmed morphene habitue. It will assist us in forming a prognosis to notice the effects of the narcotic on the system. It is primarily that of a stimulant, heightening the functional activity of the several organs; and secondarily that of a depressent to those same organs. In either case the disturbance is usually functional, and therefore reparable as soon as the cause is removed. An important fact to be taken into account is the physical condition of the patient. If he is suffering from painful incurable disease it is not often advisable to deprive him of his narcotic unless it produces such mental gloom that the habitue longs to free himself from his bondage at any cost. In the aged the question of treatment has to be decided according to the individual case, but in general this class find their years an excuse for continueing the habit. A highly marked nervous diathesis diminishes the chance of permanent success. It is important in all cases, however, to discriminate between the symptoms produced by the narcotic and those that are caused by an independent disease. The continued use of morphia simulates many a grave malady, the symptoms of which disappear as if by magic on the discontinuance of the drug.

The efficacy of a given course of treatment is most surely demonstrated in its effecting the object for which it was instituted. So many cases of morphine inebriety have been subjected to this test and have come out completely and permanently cured, that their successful treatment has passed beyond the stage of experimentation. The consumption of large quantities of morphia even for many years presents no barier that cannot be overcome. We will give a few illustrative cases gathered from vcrious reliable sources. Dr. Albert Day gives the case of a lady aged 36, who for 13 years took morphine per orem, the maximum daily quantity being 60 grains. She was placed under suitable treatment and in a few weeks was discharged cured. That was over 20 years ago. Recently a report came from her western home that she was "well and happy," not having taken any morphine since her addiction to it. Dr. E. H. Van Densen has reported several cases as cured, and mentions the fact that Mrs. H. was "known to be faithfully holding on as late as six years from hcr discharge."

The following cases in the practice of Dr. J. B. Mattison afford additional truth to the position taken. Dr. H., ten years a morphine habitue, taking 18 grains daily hypodermically, cause peritonitis, effects constipation, vesical and sexual debility, indigestion, anorexia, hemorrhoids, mental depression, muscular weakness and emaciation. He was so much of a mental and physical wreck that he was put on a course of tonic treatment, consisting of iron strychnine and digitalis for ten days before commencing special treatment, which bcgan by reducing the opiate to six grains. In eight days the entire drug was withdrawn, the last dose being one third of a grain. He made a very rapid recovery, and was discharged cured on the thirty-first day of treatment. In a letter to the doctor some time afterwards he says, "there is not the least physical desire for morphine." He is leading an active, useful life in his profession.

The next case was also a physician, who had used morphine for the same period and in the same manner. The largest daily quantity taken was thirty grains, and the amount used at the beginning of treatment was fourteen grains After eight days of special treatment the narcotic was abandoned. The effects were restlessness, insomnia, pain in lower limbs and occasional abdominal pain. Normal sleep returned in a week, the patient rapidly improved and was discharged cured on the twenty-sixth day of treatment.

Dr. Crothers furnishes the case of Mr. B., consuming twenty-eight grains of opium for insomnia. He desired to have the opiate removed at once. It wos reduced the first day one-half, and on the third day entirely withdrawn. No serious reaction ensued. In six weeks was discharged and resumed business. He was heard from four years after and had "kept the faith."

These cases are not presented because they are unusual in any respect, but because a plain record of the results of clinical experience furnishes the most trustworthy evidence of the value of a given course of treatment.

Submitted to this test the treatment of properly selected cases of morphene inebriety is eminently successful. A cure can be predicted with more certainty than in a majority of diseases· A question or two often arises in this connection. Will the cure be permanent? Will not the patient relapse into his old habit? A few of the comparatively large number cured have "fallen from grace" but they have fallen only to rise again and with firm resolve press foward. In the end they have become victorious. Out ot a large number of cases with which we are acquainted we do not know of one that has been cured who again permanently acquired the habit.

Another point to be considered is, will it pay? What is the cost in time and suffering? The time varies greatly with the nervous organization of the patient, and the inroads made on his system by the opiate and by disease. In general, hwever, the treatment covers from one to three months. Surely any victim can afford that period to get rid of one of the most terrible habits that was ever formed. Modern treatment has nearly banished acute suffering. In some cases there is insomnia, neuralgia, diarrhea, hallucinations and delusions, a sense of exhaustion, and various other derangements. If treatment does not entirely relieve them it renders them bearable.

From the testimony taken it appears that morphine inebriety is easily curable, that relapses rarely occur, and that the expense is insignificant in comparison with the result achieved. The thing above all else is that the patient unreservedly surrenders himself to his physician obeying his rules, and carying out his suggestions as far as possible. This done, and the successful treatment of a suitable case is assured.—*Quarterly Review of Narcotic Inebriety.*

